# The Natural History of Aerosolized *Francisella tularensis* Infection in Cynomolgus Macaques

**DOI:** 10.3390/pathogens10050597

**Published:** 2021-05-13

**Authors:** Ondraya M. Frick, Virginia A. Livingston, Chris A. Whitehouse, Sarah L. Norris, Derron A. Alves, Paul R. Facemire, Douglas S. Reed, Aysegul Nalca

**Affiliations:** 1 Veterinary Medicine Division, U.S. Army Medical Research Institute of Infectious Diseases (USAMRIID), Frederick, MD 21702, USA; ondraya.m.frick.civ@mail.mil (O.M.F.); virginia.a.livingston6.civ@mail.mil (V.A.L.); sarah.l.norris2.civ@mail.mil (S.L.N.); 2 Naval Medical Research Center, Undersea Medicine Department, Silver Spring, MD 20910, USA; 3 Molecular and Translational Sciences Division, U.S. Army Medical Research Institute of Infectious Diseases (USAMRIID), Frederick, MD 21702, USA; Chris.Whitehouse@fda.hhs.gov; 4 Office of Research, Center for Veterinary Medicine, U.S. Food and Drug Administration, Laurel, MD 20708, USA; 5 Pathology Division, U.S. Army Medical Research Institute of Infectious Diseases (USAMRIID), Frederick, MD 21702, USA; derron.a.alves.mil@mail.mil (D.A.A.); paul.r.facemire2.mil@mail.mil (P.R.F.); 6 Veterinary Services and Public Health Sanitation Directorate, Army Public Health Center, Aberdeen Proving Ground, MD 21010, USA; 7 Center for Vaccine Research, University of Pittsburgh, Pittsburgh, PA 15261, USA; dsreed@pitt.edu; 8 Core Support Directorate, U.S. Army Medical Research Institute of Infectious Diseases (USAMRIID), Frederick, MD 21702, USA

**Keywords:** *Francisella tularensis*, tularemia, aerosol, animal model, cynomolgus macaque

## Abstract

Tularemia is a severe, zoonotic infection caused by the Gram-negative bacterium *Francisella tularensis*. Inhalation results in a rapid, severe bacterial pneumonia and sepsis, which can be lethal. Because the cynomolgus macaque is the accepted nonhuman primate model for tularemia, we conducted a natural history study of pneumonic tularemia by exposing macaques to target inhaled doses of 50, 500, or 5000 colony forming units (CFU) of *F. tularensis* subsp. tularensis SCHU S4. Two animals within the 50 CFU group (calculated doses of 10 and 11 CFU) survived the challenge, while the remainder succumbed to infection. Exposure of cynomolgus macaques to aerosolized SCHU S4 resulted in fever, anorexia, increased white blood cell counts, lymphopenia, thrombocytopenia, increased liver enzymes, alterations in electrocardiogram (ECG), and pathological changes typical of infection with *F. tularensis*, regardless of the challenge dose. Blood pressure dropped during the febrile phase, particularly as temperature began to drop and macaques succumbed to the disease. ECG analysis indicated that in 33% of the macaques, heart rate was not elevated during the febrile phase (Faget’s sign; pulse-temperature disassociation), which has been reported in a similar percentage of human cases. These results indicated that infection of cynomolgus macaques with aerosolized *F. tularensis* results in similar disease progression and outcome as seen in humans, and that cynomolgus macaques are a reliable animal model to test medical countermeasures against aerosolized *F. tularensis*.

## 1. Introduction

*Francisella tularensis* is an intracellular Gram-negative bacterium and the causative agent of the disease tularemia. Infection generally produces an acute febrile illness; the disease can manifest as a range of possible clinical presentations, largely dependent on the route of infection, dose, virulence of the bacterial strain, and host immune response [1]. After exposure, *F. tularensis* multiplies at the initial site of infection prior to spreading to the regional lymph nodes, liver, and spleen, and then to other organs [2]. The most common form of disease is ulceroglandular, involving an ulcer at the site of entry and regional lymphadenopathy, which is commonly acquired by direct contact with infected animals or arthropod bites [3]. Less commonly, variations of ulceroglandular disease are associated with different sites of entry and include oculoglandular and oropharyngeal disease. The oropharyngeal form results from ingesting contaminated food or water and, while rare, it has been reported with increasing frequency in Turkey and other European countries [4,5,6]. Pulmonary disease (pneumonic tularemia) can result from clinical progression of the ulceroglandular forms or from direct inhalation of *F. tularensis* organisms. Further clinical progression from any of the forms of tularemia can result in typhoidal tularemia leading to a state of septic shock, organ failure, and death of the host. *F. tularensis* is highly successful at evading the immune system, principally by escaping the phagosome and replicating in the cytosol of eukaryotic cells, including macrophages and dendritic cells. Dissemination of the microbe to distant sites within the body occurs via trafficking through the regional lymph nodes into the bloodstream [7].

Naturally occurring outbreaks of pneumonic tularemia still occur in the United States, as exemplified by outbreaks on Martha’s Vineyard, Massachusetts in the summer of 1978 and again in 2000 [8,9]. The number of cases averaged in the thousands early in the 20th century and has steadily declined since the 1950s, likely a result of increased urbanization of the population. Historically, *F. tularensis* was studied by Japan (prior to 1945) and the former Soviet Union for its potential as a biological weapon, and it was included in the United States offensive biological weapons program until that program was discontinued in 1969 [10,11]. A WHO study in 1969 found that intentional release of aerosolized *F. tularensis* in a densely populated area could cause 250,000 cases and 19,000 fatalities in a short timeframe [12]. The organism is currently listed as a Tier 1 select agent by the Federal Select Agent & Toxins Program (FSAP), those for which there is the most concern about intentional use as a biological weapon [13].

Models of tularemia disease have been evaluated in a variety of animal species, including mice, rabbits, rats, guinea pigs, and monkeys [14]. In a previous study, we compared the LD_50_ and disease outcome of inhalational tularemia in three nonhuman primate (NHP) species (cynomolgus and rhesus macaques, and African green monkeys) [15,16]. Thus, to more extensively characterize the disease progression and pathogenesis in a cynomolgus macaque model, we exposed groups of cynomolgus macaques to three different target doses (50 CFU, 500 CFU, and 5000 CFU) of aerosolized *F. tularensis* SCHU S4. Based on these data, as well as more comprehensive analysis of studies from multiple facilities, the cynomolgus macaque inhalation model was selected as the nonhuman primate model of the human disease resulting from inhalation exposure to *F. tularensis*. The resulting data from this study contributed to the knowledge base needed for use of this model for evaluation of vaccines and therapeutics for tularemia to satisfy the FDA Animal Rule.

## 2. Results

### 2.1. Survival and Clinical Signs

**Figure 1:**
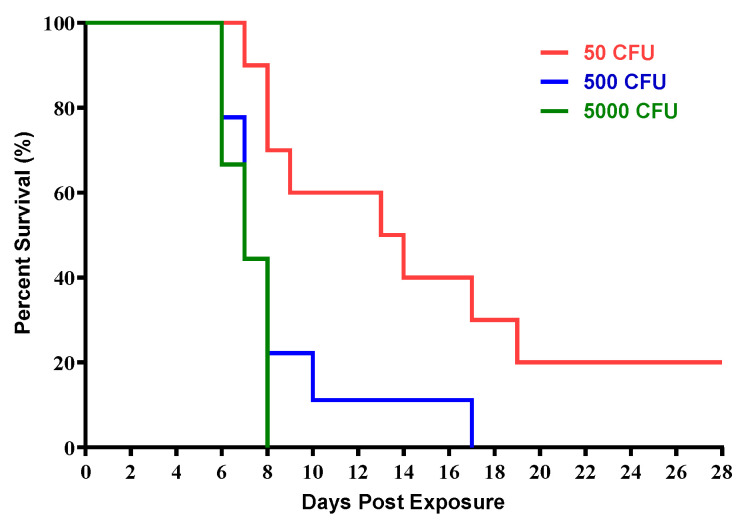
Survival of cynomolgus macaques exposed to increasing doses of aerosolized *F. tularensis*. Group sizes were *n* = 10 (50 CFU target dose), *n* = 9 (500 CFU target dose), and *n* = 9 (5000 CFU target dose).

Three groups of cynomolgus macaques were exposed to increasing doses of aerosolized *F. tularensis* SCHU S4 (Table 1). The target doses of 50 CFU (*n* = 10), 500 CFU (*n* = 9), and 5000 CFU (*n* = 9) were chosen based on *F. tularensis* SCHU S4 median lethal dose (LD_50_) in cynomolgus macaques previously determined. The calculated inhaled doses were comparable to the target doses for each group (Table 1). Survival was dose-dependent, with the exposures to high doses of 5000 CFU (range of 1177 CFU to 5860 CFU) and the intermediate dose of 500 CFU (range of 134 to 749 CFU) resulting in no surviving animals. Exposures to the low dose of 50 CFU (range of 10 CFU to 110 CFU) resulted in survival of 2 out of 10 (20%) animals which were exposed to 10 and 11 CFUs (Figure 1). There were significant differences in the survival curves when comparing the low dose group to the intermediate (*p* = 0.0324) and high dose groups (*p* = 0.0036) (Table 2). With the exception of one animal in the intermediate dose group, the majority of macaques receiving high and intermediate doses met criteria for euthanasia between days 6 and 8, whereas animals receiving low doses had a much wider range, meeting criteria for euthanasia between 7 and 19 days (Table 1). Since most of the animals succumbed to disease on day 10, the following data is showing only up to day 10 post infection pi accurate comparison.

**Figure 2:**
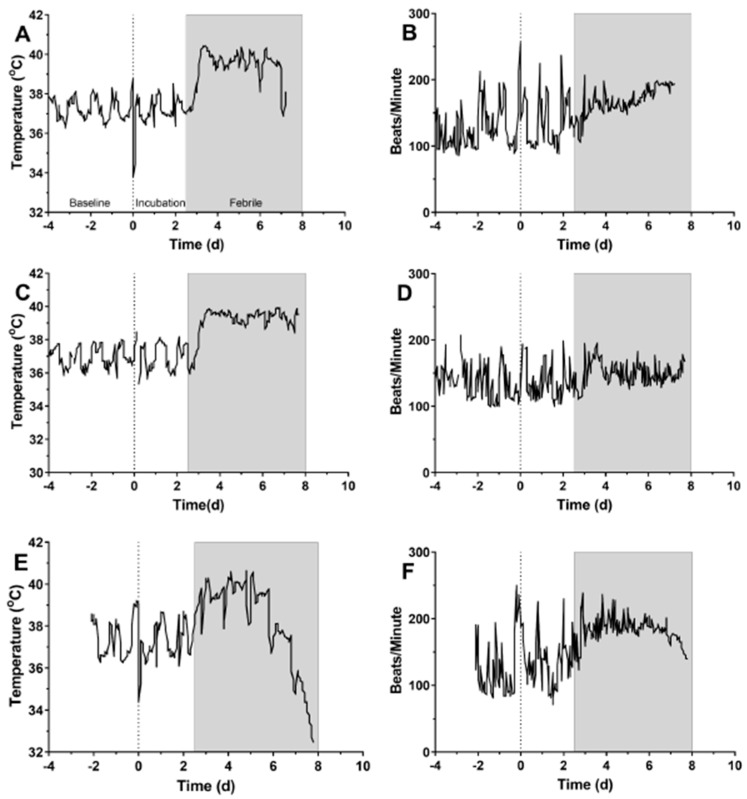
Fever and heart rate changes recorded by telemetry in macaques exposed to aerosolized *F. tularensis*. Data is shown from representative data from individual macaques in the low (**A**,**B**), intermediate (**C**,**D**), and high (**E**,**F**) dose groups. Data collected prior to infection is considered baseline and is used for prediction of body temperature post-infection (**A**). The period between infection and fever onset is considered the incubation period. The gray bar represents the febrile period in each macaque to allow comparison with the heart rate data, beginning at 2.5 days post-infection and continuing to day 8 post-infection.

Figure 2 shows temperature data from representative macaques in each dose group. Except for two macaques in the lowest dose group, all macaques exposed to *F. tularensis* in this study developed a fever between one and four days after infection. The onset of sustained (6+ hours) fever was generally earlier in the higher dose group (day 1.8 vs. day 3.4, Table 3), however, the differences in onset of sustained fever between the groups failed to meet criteria for statistical significance (*p* = 0.0644) (Table 2). Fever hours and maximum elevation in body temperature were longer and higher in the high dose group. However, there were no significant differences between groups for fever duration or average elevation (Supplementary Table S1).

**Figure 3:**
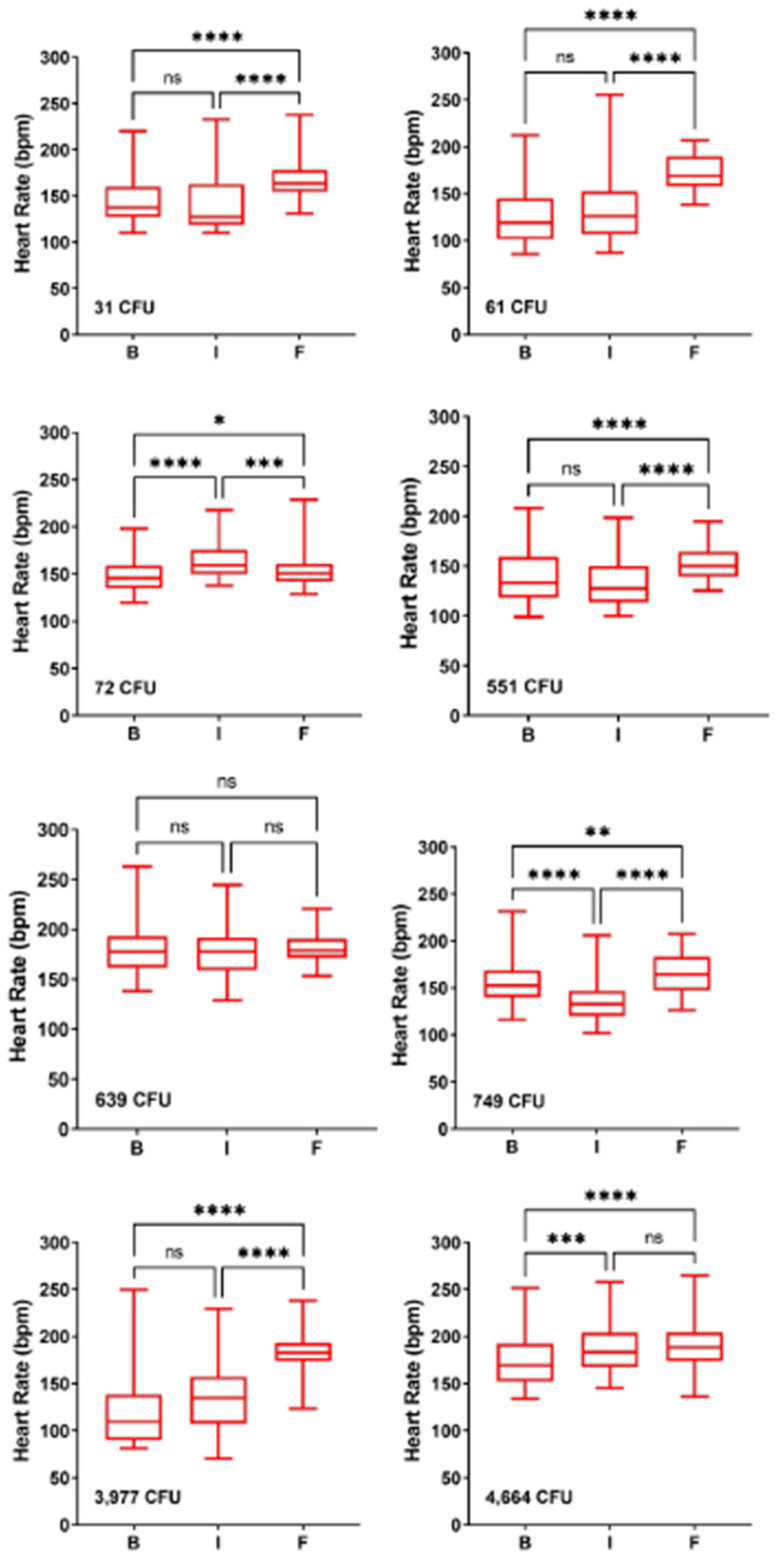
Mild elevation in heart rate during the febrile period. Data is shown from eight individual macaques across all dose ranges; values plotted are the median with the standard deviation and range for each period. Challenge doses are shown for each macaque. Significant changes from baseline were determined by Brown–Forsythe’s analysis of variance (ANOVA). n.s. = not significant, * = *p* < 0.05, ** *p* < 0.01, *** *p* < 0.001, **** *p* < 0.0001.

A subset of macaques were implanted with telemetry devices that monitored ECG and blood pressure in addition to body temperature. Temperature and heart rate data from-three representative macaques from each dose group are shown in Figure 2. In two of the three macaques, there was a clear elevation in heart rate that roughly coincides with fever; in the third macaque, the heart rate was not elevated, although there was an apparent loss in normal diurnal variation. Median heart rates in the baseline, incubation (pre-fever onset) and febrile period were analyzed further for individual macaques; the results are shown in Figure 3 and Table 4. For five of the eight macaques shown in Figure 3, there was a highly significant increase in heart rate in the febrile period; in the remaining three heart rate was not as significantly increased or was not significant at all. Further evaluation of the heart rate data found that only one of thirteen macaques did not have a significantly elevated heart rate in the febrile period (Table 4). Elevation in heart rate roughly correlated with the challenge dose (*r*^2^ = 0.49). However, the degree of elevation in the febrile period was quite low, with a median percent change of only 21.1%. Six of the thirteen macaques had a less than 20 percent increase in heart rate from baseline. Using Leibermeister’s rule of an expected 8 bpm increase in heart rate with every 1 °C increase in body temperature, the predicted increase in heart rate during the febrile period was calculated and compared with the actual increase in heart rate for each macaque (Table 4). For one macaque, we were not able to predict the increase in heart rate because of insufficient baseline temperature data. Based on the elevation in heart rate between the febrile and baseline periods, it is likely this macaque had a heart rate elevated greater than what was predicted. For eight of the macaques, the actual increase was greater than predicted in the febrile period. For four macaques, however, the actual increase in heart rate during the febrile period was lower than was predicted.

**Figure 4:**
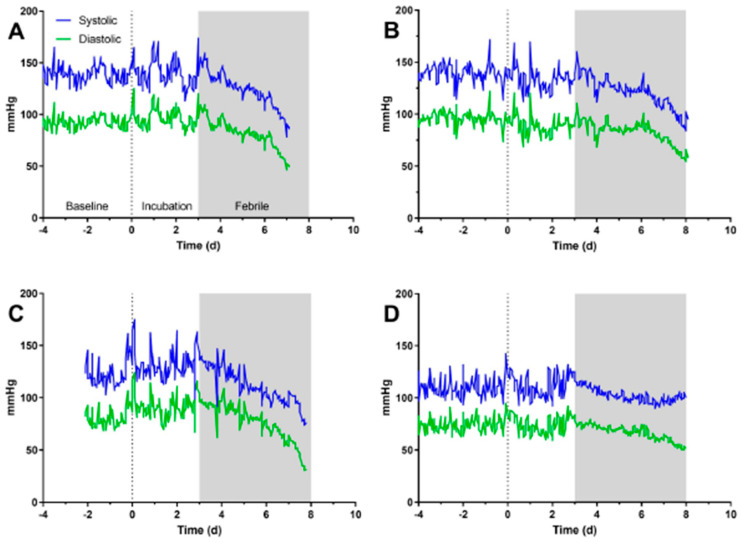
Loss of blood pressure during the febrile period. Graphs shown data for systolic (blue) and diastolic (green) blood pressure recorded by telemetry from four individual macaques (**A**–**D**) across all dose ranges. Gray bars on each graph mark the febrile period.

Figure 4A–D shows changes in blood pressure over time for four macaques infected with *F. tularensis*. Prior to fever onset, there is no notable change in either systolic or diastolic blood pressure. For three of the four, blood pressure drops throughout the febrile period until the macaque succumbed to the infection (Figure 4A–C). In the fourth animal, diastolic pressure drops in the febrile period, but systolic pressure does not (Figure 4D). There is, however, a notable change in the pattern of systolic pressure with the data overall smoother and less variation during the febrile period. It is notable that of the four shown, this macaque did not have an elevated heart rate.

**Figure 5:**
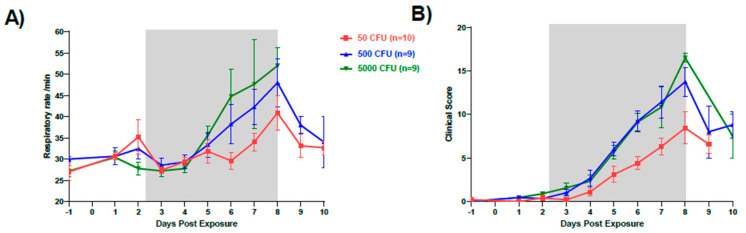
Changes in respiration and clinical score after infection. Graphs show changes in (**A**) average respiration rate and (**B**) average clinical score for each dose group. Gray bars on each graph mark the febrile period.

Increases in respiration rate were noted in all groups. While low and intermediate dose group macaques demonstrated increased respiratory rate starting on Day 3, the high dose group had an increased respiratory rate on Day 4. All macaques in the high group dose had gradually increased respiratory rates until they were euthanized (Figure 5A). Clinical signs noted included increased body temperature, elevated white blood cell (WBC) count, and increased respirations. Overall, the onset of increase in clinical score was earliest in the high dose group on Day 2 post-exposure with decreased appetite (Figure 5B). Over half of all macaques developed a cough between Days 7 and 9 with the greatest observed numbers in the high dose group (78%). Loose stool was observed primarily in the low dose group and in 25% of macaques overall. Body weights of all macaques were taken on Day-1 and on the day of euthanasia. The high and intermediate dose groups showed similar weight loss of 8.3% (*p* = 0.0171) and 8.7% (*p* = 0.0171), respectively, whereas the low dose group lost an average of 2.4% (data not shown). Disease scores for all groups exposed to *F. tularensis* peaked on Day 8 post-exposure and the last members of the high dose group macaques were euthanized on this day.

### 2.2. Clinical Laboratory Evaluation

**Figure 6:**
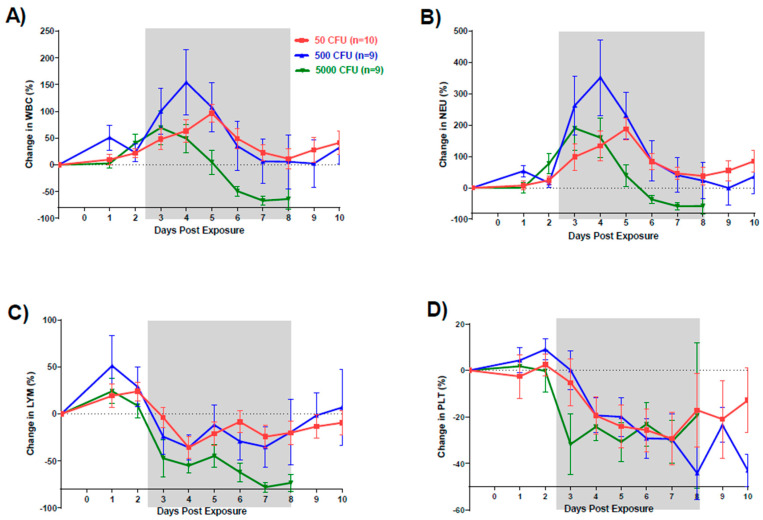
Changes in white blood cells after infection (suggested). Graphs show changes in average cell counts by dose group for (**A**) total white blood cells, (**B**) neutrophils, (**C**) lymphocytes, and (**D**) platelets.

**Figure 7:**
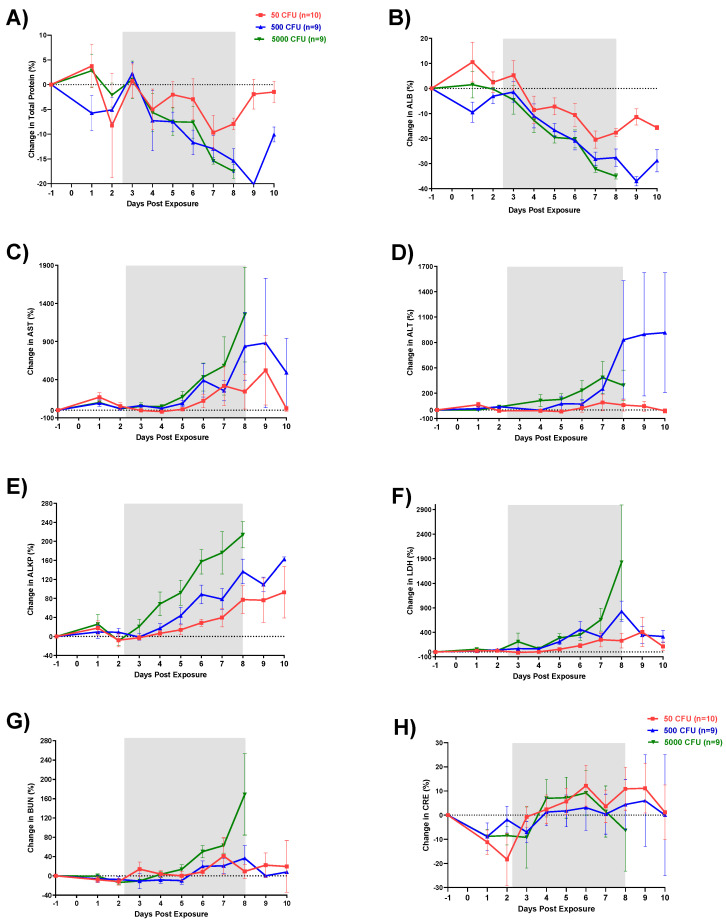
Changes in serum chemistry parameters in cynomolgus macaques exposed to different doses of aerosolized *F. tularensis*. (**A**) Total protein, (**B**) albumin, (**C**) aspartate transaminase (AST), (**D**) alanine transaminase (ALT), (**E**) alkaline phosphatase (ALKP), (**F**) LDH (lactate dehydrogenase), (**G**) blood urea nitrogen (BUN), (**H**) creatinine (CRE). Blood chemistries were analyzed with VITROS 250 chemistry analyzers.

Changes in CBCs and blood chemistries were observed in all groups after exposure to *F. tularensis*. Similar to our observations in the previous published study, WBC counts increased starting Day 2 for the high and low dose group and Day 3 for the intermediate dose group (Figure 6). Decrease in total protein and albumin (ALB) values observed by Day 3 (Figure 7A,B). There were no significant differences in total protein between the three groups. In contrast, albumin values were significantly lower in the intermediate (*p* = 0.0003) and high dose groups (*p* = 0.0014) compared to the low dose group. In addition, both intermediate and high dose group values were significantly lower than the normal range (*p* < 0.05). Liver enzymes aspartate transaminase (AST), alanine transaminase (ALT), and alkaline phosphatase (ALKP) were elevated in all groups (Figure 7C–E). Pairwise comparisons showed significant differences between the low and high dose groups for AST, ALT, and ALKP (*p* = 0.0062, *p* = 0.0118, and *p* < 0.0001, respectively). The greatest increases outside of the normal range were observed in elevated ALKP values for the high dose group on Days 6 (*p* < 0.001), 7 (*p* < 0.01) and 8 (*p* < 0.001). Levels of LDH (lactate dehydrogenase) increased starting on Day 4 for all groups with the high dose group showing the greatest increases in comparison to the low dose group (*p* = 0.0385) (Figure 7F). Except the highest dose group, blood urea nitrogen (BUN) levels stayed in normal limits; similarly, creatinine (CRE) levels remained within a low/normal range (Figure 7G,H).

### 2.3. Pathology

Postmortem examinations (necropsies) were performed on all euthanized macaques for macroscopic review with complete sets of tissues collected for microscopic analysis. The macroscopic lesions observed in this study were similar to those previously reported in cynomolgus macaques following aerosol exposure to *F. tularensis* SCHU4 regardless of the dose received, and/or the time-to-death following *F. tularensis* exposure. The most notable lesions were observed in the thoracic cavity, mainly in the lungs and mediastinal and tracheobronchial lymph nodes.

**Figure 8:**
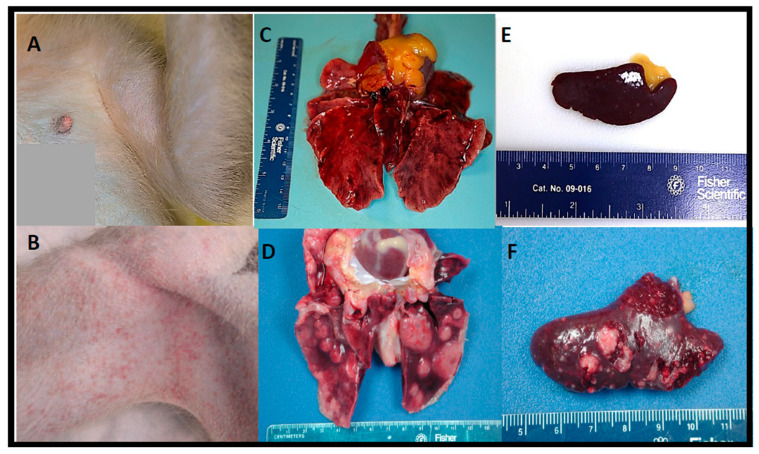
Comparison between nonchallenged control cynomolgus macaques and the gross pathological changes noted in cynomolgus macaques exposed to aerosolized *F. tularensis*. (**A**) Axillary area with no evidence of a cutaneous rash. (**B**) Axillary area of NHP exposed to 5000 CFU. (**C**) All lung lobes appear grossly normal with the exception of postmortem congestion. (**D**) Lung from a cynomolgus macaque exposed to 5000 CFU SCHU S4; hemorrhagic, edematous, and noncollapsing lung with multiple, raised necrotizing, and/or pyogranulomatous foci on all lung lobes. (**E**) Normal sized and shaped spleen. (**F**) Spleen from a cynomolgus macaque exposed to 50 CFU; multiple, tannish-white, flattened to slightly raised foci.

During the gross postmortem examination, a focally extensive petechial rash (minimal in severity) was observed bilaterally in the axillary and inguinal areas of some of the macaques (Figure 8B). Macroscopic pulmonary changes varied in severity between macaques. Typically, all lung lobes were affected; however, lesions in the caudal (inferior) lung lobe were generally more severe. One or more of the following pulmonary changes were observed in the lung lobes: enlargement (failure to collapse); edema; mottled dark reddish-purple to black discoloration (consistent with severe pulmonary congestion, hemorrhage, and/or edema); and fibrinous pleuritis (a lesion seen more commonly on the pleural surface of the caudal (inferior) lung lobes). Fibrinous tags and adhesions loosely adhered to the thoracic wall and between the pleural surfaces of lung lobes, the diaphragm, and pericardial sac. Randomly scattered, well-circumscribed to coalescing, tannish-white, dome-shaped, firm to fluctuant foci (consistent appearance with necrosis) ranging in size from 6 mm up to 2 cm in diameter were prominent in the (caudal) lung lobes; however, similar lesions were randomly distributed in other lobes (Figure 8D).

Enlargement (lymphadenopathy) of lymph nodes with edema and reddish discoloration (suggestive of hemorrhage/congestion) were the most common macroscopic findings. The mediastinal and tracheobronchial lymph nodes were the most commonly affected lymph nodes. However, similar findings were present in the mandibular, axillary, inguinal, and mesenteric lymph nodes. Splenic changes were also common but variable between macaques. The most noteworthy macroscopic lesions consisted of few to many (too numerous to count), up to 4 mm in diameter, tannish-white, flattened to slightly raised foci randomly dispersed throughout the parenchyma and capsular surface (Figure 8F). In some cases, a normal sized to moderately enlarged (splenomegaly), dark-reddish brown turgid spleen (suggestive of diffuse congestion and/or hemorrhage) was the sole splenic finding. For comparison, refer to Figure 8A–C for gross images of the axillary region, lung, and spleen, respectively, from a nonchallenged control cynomolgus macaque.

A mildly enlarged, dark reddish brown, congested liver (hepatomegaly) with an accentuated lobular pattern was observed in half of the macaques. Occasionally, randomly distributed, 1–2 mm tannish-white foci (consistent with necrosis) were seen on the hepatic surface; similar lesions were seen on the renal capsular surface of one macaque (data not shown).

**Figure 9:**
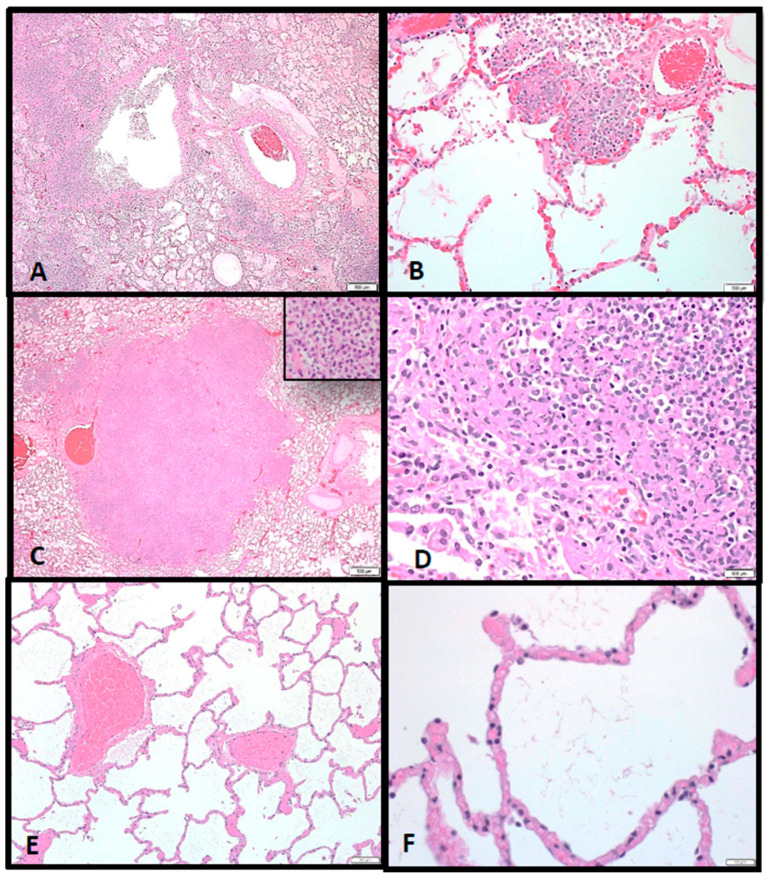
(**A**) Multifocal to coalescing foci of inflammation, often associated with larger conducting airways and arterioles, causing severe pulmonary consolidation (pneumonia), 2×. (**B**) Alveolar inflammation composed of neutrophils and fewer macrophages is seen during initial infection, 10×. (**C**) A well-circumscribed chronic abscess effaces pulmonary architecture in the 7–9 dpi animals, 20×; Inset. Chronic abscesses consist of degenerate neutrophils and fewer macrophages. (**D**) Abscesses become more widespread with significant pyogranulomatous inflammation characterized epithelioid macrophages with foamy vacuolated cytoplasm and fewer degenerate neutrophils at 14–17 dpi, 40×. (**E**) Normal alveolar airs spaces adjacent to arterioles from an aged match, nonchallenged NHP, 10×. (**F**) No histologic evidence of inflammation present in the alveolar air spaces at higher magnification, 40×.

Almost all macaques had noteworthy microscopic lesions specifically in the lung, lymph nodes, spleen, and bone marrow, which correlated well with the macroscopic findings described above. Lesions were similar in all macaques but varied in severity. Microscopic findings in the lungs consisted of necrotizing and suppurative bronchopneumonia (associated with larger conducting airways and arterioles) or randomly distributed vague to discrete inflammatory nodules (consistent with chronic abscesses and pyogranulomatous pneumonia) (Figure 9A–D). Intra-alveolar inflammation composed of neutrophils and macrophages were the earliest findings in all animals (Figure 9B). Macaques that expired at Days 7–9 post-exposure had lesions consistent with chronic abscesses, whereas those expiring at Days 14–17 dpi had more widespread pyogranulomatous inflammation (Figure 9C,D, respectively). Immunolabeled intra- or extracellular bacteria, bacterial fragments, and/or antigen were commonly seen in foci of necrosis in those tissues examined. Figure 9E,F show normal lung areas.

## 3. Discussion

Respiratory or inhalational infection with aerosolized *F. tularensis* in humans causes a rapid, severe bacterial pneumonia and sepsis which can be lethal with a minimum infectious dose of fewer than 10 CFU [17]. Due to its high infectivity and relative ease of dissemination, *F. tularensis* has been studied as a potential candidate for weaponization and categorized as an agent of biological terrorism [2,9,10]. Development of medical countermeasures to lethal diseases such as inhalation tularemia is contingent on the characterization of animal models that effectively mimic the most severe form of disease in humans. The cynomolgus macaque has been established as the nonhuman primate model for pneumonic tularemia. To further characterize the disease in the cynomolgus macaque, groups of cynomolgus macaques were exposed to target doses of 50, 500 or 5000 CFU of aerosolized *F. tularensis*. Survival was dose dependent with target doses of 500 CFU (134 to 749 CFU range) and 5000 CFU (1177 to 5860 CFU range) causing 100% lethality by Days 7 and 8, respectively. Target doses of 50 CFU (10 to 110 CFU range) resulted in survival of 2 out of 10 macaques (20%).

In humans, tularemia is an acute febrile illness. The disease manifestation is dependent on the route of infection, the dose, virulence of the strain, and host immune response. After an average incubation period of 3–5 days, there is an onset of symptoms including fever, chills, cough, headache, general malaise and in some cases diarrhea [18,19]. In this study, fever began in cynomolgus macaques generally between 2–3 after aerosol exposure to *F. tularensis*. Onset of other clinical changes noted (heart rate, blood pressure, changes in behavior, hematological changes) all coincided with fever onset. This data is strikingly similar to what has been reported in rats and rabbits, particularly with regard to fever onset and other clinical signs [20,21].

The generally mild increase in heart rate seen in most macaques was quite intriguing. While heart rate was elevated compared to baseline in 12 of 13 macaques, the heart rate in the febrile period was still within the normal range for an unrestrained adult cynomolgus macaque [22]. In other severe zoonotic infections in nonhuman primate models, heart rate increases between 40–60% in the febrile period (D.S. Reed, manuscript in preparation). This contrasts sharply with the median 20% increase seen here, and notably six of the thirteen macaques had a less than 20% increase in heart rate. Leibermeister’s rule stipulates an 8 bpm increase in heart rate for every 1oC elevation in body temperature [23]. This allowed us to calculate a predicted increase in heart rate during the febrile period; while 8 of 12 had an increase in heart rate greater than predicted, for four macaques (33%) heart rate was lower than predicted. Intriguingly, Faget’s sign (fever paired with bradycardia) has been reported in 42% of human tularemia patients [24]. Faget’s has also been reported in yellow fever, typhoid, brucellosis, and Legionnaire’s disease. This finding lends further credence to the cynomolgus macaque as a relevant model of human tularemia and would further suggest that this model could be used to gain a better understanding of the underlying pathophysiology associated with Faget’s sign.

Hematology and clinical chemistry parameters in humans infected with *F. tularensis* have not shown any consistent remarkable findings. A review of 88 cases of tularemia from 1949 through 1979 reported increased white blood cell counts ranging from 5000 to 22,000 cells/mm^3^ (median 10,400 cells/mm^3^) upon hospital admission. An additional review of over 300 of cases reported that 50% presented with WBC counts between 3000 and 24,000 cells/mm^3^ however the other half showed marked increases in leukocytes as great as 56,000 cells/mm^3^ [22]. Similarly, in this study, there was a clear increase in WBC by Day 3 followed by a sharp decrease between Days 4 and 6 for all macaques; however, no group fell significantly outside of the normal range. This decrease was attributable to a loss in lymphocytes. A similar loss in platelets was also seen on Day 3. These changes in CBC match very well with what has been reported in both rats and rabbits. Increases in liver enzymes AST, ALT, and ALKP, as seen in this study, have been previously reported in human tularemia cases where of 58 patients tested, 58% had elevations in at least one of these three enzymes.

As previously seen in lethal human cases of tularemia [25], in this study, the lung, lymph nodes (particularly the mediastinal and tracheobronchial lymph nodes), spleen, liver, and bone marrow were the most severely affected tissues during *F. tularensis* infection regardless of exposure dose. Although varying in severity, multifocal and coalescing, necrotizing and pyogranulomatous pneumonia and abscessation with (or without) extensive edema, fibrinous pleuritis, and pleural fibrosis were consistent pathological findings in almost all macaques and corresponded with the intrathoracic histopathologic changes. Additionally, necrotizing mediastinal lymphadenitis, splenitis, myelitis, and hepatitis were consistently observed regardless of the inhaled *F. tularensis* dose. The random focal necrotizing lesions in the liver were discrete but small unlike the larger coalescing lesions seen in the lung and spleen.

Here we demonstrated that aerosolized *F. tularensis* infection in cynomolgus macaques causes similar disease progression, pathogenesis, and clinical pathology profiles as compared to respiratory tularemia in humans. Therefore, the cynomolgus macaque model of pneumonic tularemia is an appropriate animal model for testing medical countermeasures for treatment of tularemia.

## 4. Materials and Methods

### 4.1. Animals and Ethical Statement

Twenty-eight healthy, adult female and male cynomolgus macaques (Macaca fascicularis) of primarily Chinese descent weighing between 3.6 kg and 7.4 kg were obtained from USAMRIID NHP colony. The age of the animals ranged from 3 years to 7 years. Animals were experimentally naïve with the exception of four animals that were either previously used as negative controls or survived a previous challenge with *Francisella tularensis* SCHU S4 and had a calculated exposure dose of 0 CFU with no signs of the disease and no antibodies against *F. tularensis*. Animals were in good physical condition, lacked physical malformations, and were free of clinical signs of infectious, contagious, or communicable diseases or parasites. All animals exposed to *F. tularensis* were handled in a BSL-3 containment laboratory at the U.S. Army Medical Research Institute of Infectious Diseases (USAMRIID). Research was conducted in compliance with the Animal Welfare Act and other federal statutes and regulations relating to animals and experiments involving animals, and adhered principles stated in the Guide for the Care and Use of Laboratory Animals, National Research Council, 1996. The facility where this research was conducted (USAMRIID) is fully accredited by the Association for the Assessment and Accreditation of Laboratory Animal Care International. Research was conducted under a protocol approved by the Institutional Animal Care and Use Committee (IACUC) at USAMRIID. All animals were examined and evaluated twice per day by study personnel. Early endpoint criteria, as specified by the score parameters within the “Post-exposure observations” section of these methods, were used to determine when animals should be humanely euthanized.

### 4.2. Bacteria

*F. tularensis* SCHU S4 strain (Biodefense and Emerging Infections Research Resources Repository (BEI Resources) Cat. No. NR-10492) was provided by the National Institute of Allergy and Infectious Diseases. A 25-mL flask of Mueller Hinton II (MHII) liquid medium containing 2% IsoVitaleX enrichment was inoculated with 10 µL of a SCHU S4 seed stock. After 23 h of incubation at 37 °C with shaking at 200 rpm, the optical density (OD660 nm) was measured. Logarithmic growth cultures were at the mid-logarithmic phase of growth and had an optical density (OD660 nm) of 0.15 to 0.85. Microorganisms were diluted to the desired nebulizer concentration in MHII medium.

### 4.3. Aerosol Exposures

Each macaque was anesthetized by intramuscular (i.m.) injection of tiletamine/zolazepam (3 mg/kg) and challenged by aerosol as previously described [26]. Briefly, the respiratory function of each of the macaques was measured using whole-body plethysmography (Buxco Systems, Sharon, CT) before aerosol challenge. Aerosol procedures were conducted using a 32-L, airtight Lexan chamber assembled in a head-only configuration for individual macaque exposures in a class III biological safety cabinet located inside a BSL-3 suite. All airflows, environmental monitoring, and system balancing were configured, controlled, and recorded through the institute standard automated bioaerosol exposure system (ABES)(Biaera Technologies, LLC, Hagerstown, MD [27]. Integrated air samples were obtained for each individual exposure using an all-glass impinger (AGI) drawing 6 L/min from a port centered on the chamber and opposite to the animal’s head. Aerosols were generated with a three-jet Collison nebulizer running at 7.5 L/min, 25–30 psi. The amounts of live bacteria in AGI samples were analyzed on the same day as the aerosol exposure by performing a bacterial plating assay on Modified Thayer–Martin (MTM) agar plates to determine the colony forming units (CFU)/mL.

### 4.4. Telemetry

Macaques were implanted with a radiotelemetry device [Data Sciences International (DSI), St. Paul, MN] at least 14 days before aerosol exposure. Body temperatures, electrocardiography, and blood pressure were recorded once per hour by the DataQuest A.R.T. 4.1 system (DSI). Fever was defined as body temperature greater than two standard deviations above baseline temperature as determined by autoregressive integrated moving average (ARIMA) modeling. Due to telemetry equipment failure, two animals in the high target dose group had shorter baselines starting Day -1.5 and Day -0.5; for these animals, temperature recordings were initiated at approximately 12:00 PM on respective days.

### 4.5. Post-Exposure Observations and Clinical Scoring

Macaques were observed at least twice a day after aerosol exposure and scored for clinical signs of disease prior to, and while under anesthesia. The scoring parameters were as follows, appearance (0: normal with coat smooth, eyes/nose clear; 1: reduced grooming; 2: dull/rough coat, ocular/nasal discharge; 3: absence of grooming, or piloerection, or posture change), appetite (0: normal; 1: decreased biscuit intake but still eats enrichment × 24 h; 2: will only eat food enrichment × 48 h; 3: anorexic × 48 h), clinical signs (0: normal temperature and respiration/min; 1: Slight change in temperature/respiration (±1 °C out of normal range/< 30% ± *n* respiration range); 2: Temp: ±2 °C; respiration: ±30% (above 51 or below 27); intermittent tremors; intermittent convulsions <10 min duration; transient prostration <1 h; 3: Temp: ±3 °C; respiration: ±50% (above 59 or below 19); continuous tremors or convulsions; persistent prostration ≥1 h), natural behavior (0: normal; 1: minor changes, less interaction with staff; 2: little interaction, less mobile and alert, isolated; 3: no interaction, vocalization, self-mutilation, restless, or still), provoked behavior (0: normal; 1: subdued but normal when stimulated, 2: subdued even when stimulated, 3: unresponsive when stimulated, weak, pre-comatose). An extra point for each score of 3 was added to the cumulative clinical score. The early endpoint criteria for humane euthanasia, indicative of very poor health status, were cumulative clinical scores of 16–20 (maximum score), and/or a sudden drop of >3 °C from baseline body temperature, continuous tremors or convulsions lasting ≥10 min duration, persistent prostration lasting ≥2 h, comatose or loss of consciousness. In some cases, such as accelerated dropping in body temperature without anesthesia, severe respiratory distress or continuous tremors, where the scoring range did not reflect the severity of the disease, the decision for euthanasia was made by the study director after consultation and verbal confirmation with the attending veterinarians.

### 4.6. Clinical Laboratory Evaluation

Blood samples were collected from central venous catheters (CVC) inserted in the internal jugular vein beginning fourteen days before, one day before, Days 1–21, and Day 28 after exposure. If collection from the CVC was not possible, blood samples were collected from the femoral vein of macaques anesthetized with 3 mg/kg intramuscular tiletamine/zolazepam. Samples collected one day prior to exposure served as a reference baseline for each animal. Complete blood counts (CBCs) and blood chemistries were analyzed with Beckman Coulter hematology and VITROS 250 chemistry analyzers, respectively.

### 4.7. Postmortem Examination (Necropsy)

A full postmortem examination was performed under direct supervision of a pathologist board certified by the American College of Veterinary Pathologist in a BSL-3 necropsy facility. Tissues samples from all major organ systems (respiratory, gastrointestinal, genitourinary, hematopoietic, nervous, endocrine, skin and mucous membranes) were collected from each animal for histopathological and, in some cases, immunohistochemical examination. Tissue samples were frozen at −70 °C until processed for bacterial assays. All other tissues were immersion-fixed in 10% neutral buffered formalin for a minimum of 21 days prior to removal from biocontainment.

### 4.8. Histology and Immunohistochemistry

Formalin-fixed tissues for microscopic examination were trimmed, processed, and embedded in paraffin according to established protocols [28]. Embedded tissues were sectioned at 5–6 µm on a rotary microtome, mounted on glass slides, and stained with hematoxylin and eosin (H&E). Tissues evaluated by immunohistochemistry were stained for *F. tularensis* lipopolysaccharide (LPS) (Meridian Life Science, Inc., Cincinnati, OH, USA) using a mouse monoclonal antibody (USAMRIID immuno #927) and an immunoperoxidase assay system (EnVision System, DAKO Corp., Carpinteria, CA, USA). Unstained tissue sections were deparaffinized, rehydrated, subjected to methanol-hydrogen peroxide block, rinsed, and pretreated with proteinase K for 6 min at room temperature. A serum-free protein block (DAKO) plus 5% normal goat serum was applied for 30 min. The monoclonal antibody was then applied to the tissue at a dilution of 1:1200 and incubated at room temperature for 60 min. The tissue was exposed to the EnVision horseradish peroxidase labeled polymer for 30 min at room temperature. All sections were exposed to 3,3′-diaminobenzidine (DAB) permanent chromogen for 5 ± 1 min, rinsed, counter-stained with hematoxylin, dehydrated, and cover-slipped with Permount. Normal uninfected lung tissue served as the negative control; the positive control tissue was lung tissue from a known *F. tularensis*-infected monkey. Normal mouse IgG was used as the negative serum control for the control slides.

### 4.9. Statistical Analysis

Survival curves were calculated by the Kaplan–Meier product-limit method. Body temperatures were recorded by telemetry once per hour, beginning at least 12 h and up to 3 days prior to exposure and continuing up to 28 days post-exposure. Pre-exposure temperature data taken between two to five days pre-exposure was used to develop a baseline period to fit an ARIMA model. Forecasted values for the post-exposure periods were based on the baseline extrapolated forward in time using SAS ETS (version 9.3). For body temperature, residual changes greater than two standard deviations were used to compute fever duration (number of hours of significant temperature elevation), fever hours (sum of the significant temperature elevations), and average fever elevation (fever hours divided by fever duration in hours). The number of surviving animals in each group was statistically insufficient at later time points; therefore, only post-exposure data through Day 8 was used for analysis. T-tests with stepdown bootstrap adjustment were utilized to compare mean temperature variables between groups. ANOVA at each individual time point was completed with post hoc Tukey’s tests for pairwise comparisons for respiratory rates, CBCs and chemistries for time points Day 0 through Day 8. Beyond time point Day 8, there were insufficient samples for comparison. For analysis of ECG and blood pressure data, data were imported into Ponemah 6.5 (DSI) and analyzed using MatLab 2020a and GraphPad software.

## Figures and Tables

**Table 1:** Comparison of survival between groups.

Target Exposure Dose Groups	Average Inhaled Dose (CFU)	Days to Death *		Survivals/Total
		Mean	Std. Dev.	
50 CFU	49 ± 32	11.9	4.5	2/10
500 CFU	507 ± 250	8.4	3.4	0/9
5000 CFU	4047 ± 1563	7.1	0.9	0/9

* Nonsurvivors.

**Table 2:** Survival Comparison (*p* values).

Comparison	Time-to-Death (Days)	Survival Curve
**50 vs. 500 CFU**	0.2117	**0.0324**
**50 vs. 5000 CFU**	0.0630	**0.0036**
**500 vs. 5000 CFU**	0.2891	0.4228

**Table 3:** Summary of fever data.

	50 CFU ^e^			500 CFU ^f^			5000 CFU ^g^		
Variable	N	Mean	SD	N	Mean	SD	N	Mean	SD
Study Day of 6+ Hours of Fever ^a^	6	3.4	1.50	5	3.4	0.11	7	1.8	1.24
Days with 12+ Hours of Fever ^b^	6	3.5	0.84	4	4.5	1.00	5	3.4	1.82
Maximum Change in Temperature	8	4.6	1.70	8	3.8	2.04	7	6.3	5.39
Fever Duration (hours)	8	77.5	42.85	8	58.4	53.74	7	74.1	47.85
Fever Hours ^c^	8	246.3	165.97	8	226.8	268.24	7	447.9	450.46
Average Elevation ^d^	8	3.3	1.48	8	3.0	1.56	7	4.7	4.14

^a^ Defined as the first day with 6 or more hours of significant temperature elevation (as determined by autoregressive integrated moving average (ARIMA) modeling). ^b^ Calculated as the number of days with 12 or more hours of significant temperature elevation. ^c^ Calculated as the product of each significant increase in temperature elevation (in degrees) multiplied by the length of time of elevation (in hours). ^d^ Calculated by dividing fever hours by fever duration in hours. ^e^ Of the 10 animals in the 50 CFU group, 2 had no telemetry data, and 2 did not have 6+ hours of sustained fever. ^f^ Of the 9 animals in the 500 CFU group, 1 had no telemetry data, 3 did not have 6+ hours of sustained fever, and 4 did not have 12+ hours of sustained fever. ^g^ Of the 9 animals in the 5000 CFU group, 2 had no telemetry data, and 2 did not have 12+ hours of sustained fever.

**Table 4:** Changes in heart rate in individual macaques after infection.

Dose *	Baseline ^£^	Incubation	Febrile	Percent Change	*p*	Predicted ^γ^	Actual ^α^	Diff. ^β^
31	137.3	127.2	163.7	19.2	<0.0001	20.76	26.4	5.64
59	134.2	141.8	178.8	33.2	<0.0001	18.85	44.6	25.75
61	119	126.4	169.2	42.2	<0.0001	20.07	50.2	30.13
72	145.9	159.5	150.7	3.3	0.043	19.68	4.8	**−14.88**
551	133.4	127.5	150.1	12.5	<0.0001	19.31	16.7	**−2.61**
636	144.3	164.1	182.4	26.4	<0.0001	12.85	38.1	25.25
639	177.6	177.7	178.9	0.7	0.861	18.86	1.3	**−17.56**
728	138.2	145.6	167.4	21.1	<0.0001	12.89	29.2	16.31
749	152.5	133	164.5	7.9	0.002	13.24	12	**−1.24**
3977 ^ς^	109.4	134.8	182.8	67.1	<0.0001			
4664	169.4	183.6	188.6	11.3	<0.0001	14.93	19.2	4.27
4717	143.7	155.8	185.6	29.2	<0.0001	12.91	41.9	28.99
4894	128.7	130.1	164.4	27.7	<0.0001	20.04	35.7	15.66
**Median**	**138.2**	**141.8**	**169.2**	**21.1**		**18.9**	**27.8**	**10.6**

* dose in cfu. ^£^ Median beats per minute. ^γ^ predicted elevation in heart rate, determined by median elevation in temperature. ^α^ actual elevation in heart rate in febrile period from baseline. ^β^ difference between actual and predicted elevation in heart rate. ^ς^ predicted heart rate could not be calculated for this macaque because of inadequate baseline data for temperature forecasting.

## Data Availability

Data available on request due to restrictions and clearance processes in USAMRIID.

## Supplementary Materials

The following are available online at https://www.mdpi.com/article/10.3390/pathogens10050597/s1, Table S1: No significant differences in fever between dose groups.
