# Supplementary material for: The Natural History of Aerosolized Francisella tularensis Infection in Cynomolgus Macaques
Source: Pathogens. 2021 May 13;10(5):597. doi: 10.3390/pathogens10050597 (PMC8153158; doi:10.3390/pathogens10050597)
Supplement: Supplementary file 1 [file pathogens-10-00597-s001.zip › pathogens-1151043 supp.pdf]

pathogens MDPI

Supplementary Table S1. No significant differences in fever between dose groups.

Variable	Comparison		
	50 CFU vs. 500 CFU	50 CFU vs. 5000 CFU	500 CFU vs. 5000 CFU
Study Day of 6+ Hours of Fever	0.9666	0.0644	0.0644
Days with 12+ Hours of Fever	0.4397	0.9048	0.4397
Maximum Change in Temperature	0.6521	0.5174	0.3410
Fever Duration (hours)	0.7065	0.8941	0.7480
Fever Hours	0.8983	0.3681	0.3681
Average Elevation	0.7825	0.5138	0.4239
